# From zoonotic spillover to endemicity: the broad determinants of human coronavirus tropism

**DOI:** 10.1128/mbio.02437-25

**Published:** 2025-09-22

**Authors:** Saskia Westhoven, Luca D. Bertzbach, Mara Kloehn, Cedric Mahncke, Natalie Heinen, Richard J. P. Brown, Stephanie Pfaender

**Affiliations:** 1 Research Unit Emerging Viruses, Leibniz Institute of Virology (LIV) 28367, Hamburg, Germany; 2 Department for Molecular & Medical Virology, Ruhr University Bochum 717200, Bochum, Germany; 3 Department of Translational and Computational Infection Research (TRACiR), Medical Faculty, Ruhr University Bochum 9142, Bochum, Germany; 4 Institute of Virology and Cell Biology, University of Lübeck 237099, Lübeck, Germany; The Ohio State University, Columbus, Ohio, USA

**Keywords:** ACE2, APN, DPP4, HCoV-229E, HCoV-HKU1, HCoV-NL63, HCoV-OC43, MERS-CoV, SARS-CoV, SARS-CoV-2, spike, TMPRSS2

## Abstract

Given the recurring threat of coronavirus outbreaks, understanding the specificity of coronaviruses in terms of their host, tissue, and cell tropism is crucial. This review consolidates and critically assesses the current literature on the tropism of endemic, epidemic, and pandemic coronaviruses. We explore different levels of tropism, including species tropism (virus preference for specific host species), host cell tropism (virus specificity for particular cell types), and tissue tropism (specificity for certain tissues or organs). This review compiles extensive basic research, particularly from recent years, to provide critical insights into the viral mechanisms that are key to improving future pandemic preparedness.

## INTRODUCTION

Coronaviruses (CoVs) have repeatedly demonstrated a remarkable ability to emerge, evolve, and adapt to new hosts, posing significant and persistent challenges to global public health (1). The recent rapid spread of pathogenic CoVs underscores the need for a comprehensive understanding of viral tropism at cellular, tissue, and host levels. This understanding is crucial for predicting potential zoonotic spillovers and for mitigating future outbreaks.

By examining both receptor-dependent and receptor-independent mechanisms, the field has uncovered a complex array of strategies employed by CoVs to enter and infect host cells. These mechanisms are often influenced by viral mutations, which allow for efficient recognition and binding to host cell receptors, facilitating the ability of CoVs to infect various tissue types (2). Furthermore, adaptations that enable cross-species transmission, particularly from animals to humans, have been pivotal for the emergence of new CoVs.

Viral tropism is determined by several factors, including the presence of specific host cell receptors, the ability of the virus to enter and replicate within the host cell, the availability of necessary cellular machinery, and host immune responses. While the latter plays a critical role in shaping both tropism and species specificity, this is thoroughly discussed elsewhere and not within the scope of this review (3). Consequently, this review focuses on viral entry and, specifically, the presence of host cell receptors as a key determinant of cellular susceptibility, with the aim to build on the most recent advances in the field of CoV tropism by summarizing key findings to provide a comprehensive overview of the factors that drive viral emergence, evolution, and pathogenesis. By enhancing our understanding of these mechanisms, we can better prepare for future pandemics and develop strategies to reduce the impact of emerging CoVs on global health.

## HUMAN-INFECTING CoVs

**Fig 1:**
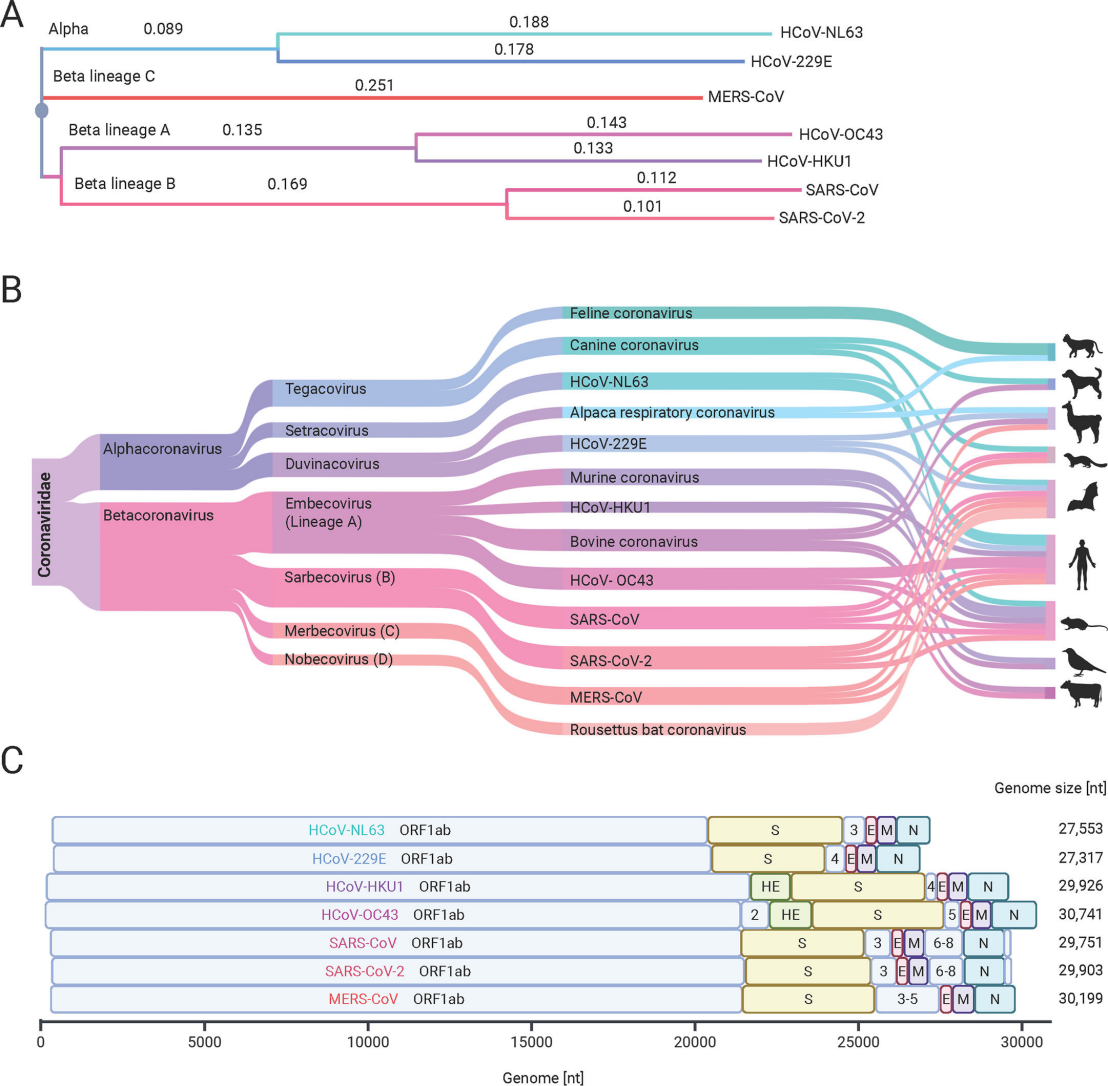
Comparative analysis of coronaviruses. (**A**) The phylogenetic tree was created from a multiple sequence alignment performed with EMBL-EBI sequence analysis tool (6). It depicts evolutionary relationships between the human-infecting CoVs among the alphacoronaviruses (blue) and betacoronavirus lineages (purple, red). Phylogenetic distances are written below the respective branch. (**B**) The Sankey plot demonstrates divergence among various CoVs and highlights common hosts (7). (**C**) The linear genomic organization of HCoVs and the position of encoded proteins highlights structural similarities and differences (8). Nonstructural protein encoding genes are presented as open reading frames and colored light blue, spike protein encoding genes are colored yellow, envelope protein encoding genes are colored red, membrane protein encoding genes are colored purple, and nucleocapsid protein encoding genes are colored green. The figure was created with BioRender.com.

To date, seven CoVs have been reported to infect humans. Human CoVs (HCoVs), belonging to the order Nidovirales, can be classified into two main groups: alphacoronaviruses and betacoronaviruses. The latter is further subdivided into four distinct lineages (A–D), reflecting their genetic composition and evolutionary relationships (Fig. 1A and B). This classification highlights the complex evolutionary dynamics of CoVs, which have emerged from various host species over time. Diverse HCoVs share a similar overall genome organization, with large, positive-sense RNA genomes encoding nonstructural proteins in the 5′ region and structural and accessory proteins in the 3′ region (4, 5). However, the number and sequence of accessory genes vary between HCoV species, reflecting differences in host interactions and pathogenicity (Fig. 1C).

In 1966, HCoV-229E was the first human CoV identified (9). This endemic alpha-CoV generally causes mild upper respiratory tract infections and is globally distributed (10). Just 1 year later, the beta-CoV HCoV-OC43 was identified in the United States (11). In 2004, two additional HCoVs were identified: HCoV-NL63 and HCoV-HKU1. HCoV-NL63, an alpha-CoV, was first detected in a child in the Netherlands and is associated with mild to moderate respiratory infections. It is particularly prevalent in children and has been linked to croup (12). HCoV-HKU1, a beta-CoV, was discovered in Hong Kong in an adult patient with pneumonia (13, 14). It typically causes mild respiratory symptoms but can also be associated with bronchiolitis and pneumonia, particularly in children or elderly individuals. All four endemic CoVs typically cause mild infections of the upper respiratory tract and are responsible for a fraction of common cold cases in adults (15). Although case fatality rates (CFRs) have not been formally defined for these viruses, they are considered low in the general population (16, 17). However, it is assumed that more severe outcomes can occur in infants, the elderly, and in immunocompromised individuals (18, 19). These viruses exhibit a seasonal and endemic circulation pattern, with widespread seropositivity indicating frequent reinfection throughout life.

In contrast to endemic HCoVs, three novel CoVs have emerged from animal reservoirs in the last two decades and are associated with more severe disease, causing significant public health emergencies. Severe acute respiratory syndrome (SARS)-CoV emerged in 2002 in the Guangdong province of China, with a high CFR of approximately 13% (20). SARS-CoV caused a global outbreak (21), spreading to over two dozen countries and resulting in more than 8,000 reported cases. The outbreak was contained in 2003 but highlighted the epidemic potential of zoonotic CoVs.

Approximately a decade later, Middle East respiratory syndrome (MERS)-CoV emerged in 2012 in Saudi Arabia, associated with severe respiratory symptoms of the lower respiratory tract in humans and a high CFR of approximately 34% with over 2,500 cases and over 900 associated deaths (22). Human-to-human transmission of MERS-CoV typically requires close contact, and prolonged transmission chains are uncommon (23). MERS-CoV continues to cause limited sporadic outbreaks, which are currently focused in the Middle East (https://www.emro.who.int/health-topics/mers-cov/mers-outbreaks.html).

The most recent addition to the group of human-infecting CoVs is SARS-CoV-2, which first emerged in Wuhan, China, in late 2019 and spread rapidly worldwide, resulting in a global pandemic. SARS-CoV-2 causes a broad spectrum of clinical presentations, ranging from asymptomatic infection to severe pneumonia and acute respiratory distress syndrome (ARDS). The CFRs vary by region, demographic factors, and healthcare capacity, with the global averages around 3.4% at the beginning of the pandemic and significantly higher rates in elderly populations and individuals with comorbidities (24). Since its initial outbreak, SARS-CoV-2 continues to circulate globally. During the course of the pandemic, multiple distinct SARS-CoV-2 variants have continuously emerged, exhibiting differences in transmissibility, immune escape potential, and pathogenicity (25). These variants are characterized by specific combinations of mutations, particularly in the spike protein, which plays a critical role in host cell entry and is a key target for neutralizing antibodies. Some variants, including alpha, delta, and omicron, rapidly supplanted the previous globally dominant strain, driven by increased infectivity and/or resistance to pre-existing immunity from prior infection or vaccination (25). Given the continuously evolving nature of SARS-CoV-2 and specifically with the emergence of omicron sublineage variants, this review differentiates between findings derived from pre-omicron variants and those specific to omicron whenever possible.

## SPECIES TROPISM

CoVs exhibit diverse host species tropism, which plays a crucial role in their pathogenesis and transmission (Fig. 1B). CoV tropism is defined by both susceptibility, determined by the presence of specific host receptors required for viral entry, and permissiveness, which refers to the capacity of a host cell to support productive infection, including the expression and activity of necessary host dependency factors. Conversely, the presence of cell intrinsic restriction factors may limit productive infection even in susceptible cells, underlining the complex interplay of host determinants that define cellular tropism (26). Different CoVs use distinct entry receptors, which vary not only in their expression across host species but also in their structural and biochemical properties. These differences, including variations in receptor-binding affinities, significantly influence host tropism and cross-species transmission potential (Table 1).

**TABLE 1:** Comparative features of HCoVs

HCoV	Host range^*a*^	Zoonotic origin^*b*^	Receptor usage	Tissue specificity in humans	Transmission efficiency
HCoV-229E	Humans, bats	Bats	APN (CD13)	Upper respiratory tract epithelium	High (endemic human transmission)
HCoV-NL63	Humans	Bats	ACE2	Upper and lower respiratory tract	High (endemic human transmission)
HCoV-OC43	Humans, cattle, pigs	Cattle	Unknown	Upper respiratory tract, possibly CNS	High (endemic human transmission)
HCoV-HKU1	Humans, rodents	Rodents (putative)	TMPRSS2	Upper respiratory tract	High (endemic human transmission)
SARS-CoV	Humans, bats, civets	Bats → civets	ACE2	Lower respiratory tract, gastrointestinal tract	Moderate (limited human to human)
MERS-CoV	Humans, camels, bats	Bats → camels	DPP4 (CD26)	Lower respiratory tract, kidneys, gastrointestinal tract	Low to moderate (limited outbreaks)
SARS-CoV-2	Humans, bats, pangolins (intermediate?)	Bats (via intermediate host)	ACE2	Upper and lower respiratory tract, multiple extrapulmonary tissues	Very high (sustained human to human)

^*a*^ Most common hosts, see also Fig. 1B.

^*b*^ Suspected.

Bats are suspected as the origin for most HCoVs, with the exception of lineage A beta-CoVs, which may have reservoirs in rodents (27). Through cross-species transmission and adaptation, these viruses have acquired the ability to infect different hosts, most notably humans, emphasizing the complexity of CoV evolution and their ability to cross species barriers.

Recent estimates indicate that the endemic HCoVs have emerged from zoonotic reservoirs within the last 1,000 years but have since undergone evolutionary adaptations, establishing humans as their primary hosts. Phylogenetic analyses suggest that HCoV-229E originated from bat CoVs (28), with transmission to humans occurring via an intermediate host, most likely camelids (29). Analysis of circulating HCoV-229E *S* and *N* genes highlights signatures of genetic drift, positive selection, and increasing divergence over time in human populations (30). Mice are resistant to HCoV-229E infection, although experimental studies suggest rodents can be made susceptible upon genetic modification (31).

The beta-CoV HCoV-OC43 is suspected to have originated from rodents (10), with bovines proposed as a likely intermediate host. A zoonotic transmission to humans may have occurred around 1890, coinciding with the “Russian flu” pandemic, although the exact causative agent—whether HCoV-OC43, influenza A virus, or another pathogen—remains uncertain to this day (32). Interestingly, a recent study revealed a close evolutionary relationship and genomic homology between HCoV-OC43 and a porcine CoV, indicating a shared evolutionary origin: this was further supported by the susceptibility of porcine intestinal organoids to HCoV-OC43 infection (33). Experimental studies have shown that HCoV-OC43 can infect mice, rapidly gaining virulence in the murine brain (34, 35). Together, these findings suggest that either pigs or rodents could potentially serve as natural hosts for ancestral HCoV-OC43-like viruses, prior to its spillover and adaptation to humans.

The animal reservoir of the alpha-CoV HCoV-NL63 remains elusive, although phylogenetic evidence suggests divergence from an HCoV-229E ancestor approximately 1,000 years ago and continued circulation in humans for centuries (36). Closely related viruses have been identified in various bat species, suggesting a potential zoonotic origin from bats (37, 38), which is further supported by permissiveness of bat cells to HCoV-NL63 infection (37). Similar to HCoV-229E, mice can be engineered to be susceptible to HCoV-NL63 infection via genetic modification (31). The most recently identified endemic circulating HCoV, HKU1, is thought to have originated from rodents (39), possibly via direct transmission to humans (40). A recent study has identified transmembrane protease serine 2 (TMPRSS2) as host receptor for HCoV-HKU1 (41) and revealed that TMPRSS2 orthologs from at least five mammalian orders including rodents support HCoV-HKU1 entry into cells (42, 43). These data support a possible rodent origin of HKU1 and implicate various species as potential reservoirs or intermediate hosts.

The initial spillover of SARS-CoV to humans highlighted the potential of CoVs to cause severe disease and initiated efforts to understand their emergence, host range, and transmission dynamics. The first SARS-CoV infections were linked to animal wet markets in China, so the hypothesis quickly arose that the virus may have crossed the species barrier, transmitting from an animal host to humans (44). Sampling of various animals sold at Chinese live animal markets for the presence of virus and neutralizing antibodies demonstrated a broad host range. Indeed, susceptibility of Himalayan palm civets, raccoon dogs, Chinese ferret badgers, hog-badgers, domestic cats, beavers, Chinese hares, and Chinese muntjacs to SARS-CoV infection was confirmed (45, 46). Studies have confirmed that the SARS-CoV epidemic lineage was likely introduced to humans via masked palm civets due to highly homologous (99.8%) viral genome identities from nasal swabs from palm civets (47), with rhinolophid bats suspected to be the zoonotic reservoir species (48–51). Since then, studies have identified multiple SARS-like CoVs in a range of bat species (48, 52–54). While bats are the likely natural source of SARS-CoV, there is still a genetic gap concerning the amplification host, in which likely recombination occurred that facilitated the species-jump toward humans. It is currently unclear where, when, and in which animal species this recombination could have occurred (55).

The first infection events of SARS-CoV-2 were epidemiologically traced to the Wuhan Huanan Seafood Wholesale Market, where various live animals were sold (56). This is further supported by a recent study tracing the genetic signature of market wildlife and SARS-CoV-2 positivity, identifying the presence of SARS-CoV-2 in stalls containing wildlife DNA from various animal species, including civets, bamboo rats, and raccoon dogs (57). Phylogenetically related viruses to SARS-CoV-2 were identified in various species, including BANAL-20-52 from Malayan horseshoe bats, RaTG13 derived from the intermediate horseshoe bat, and pangolin-CoV identified in Malayan pangolins (58–60). A recent study implied that the closest-related bat virus ancestors of SARS-CoV and SARS-CoV-2 existed less than a decade prior to their emergence in humans (61). SARS-CoV-2 can infect a wide range of hosts, including dogs, mink, ferrets, otters, hamsters, voles, deer, deer mice, bats, felines, mice, and several nonhuman primates, while the virus cannot replicate in pigs, chickens, and ducks (62–64). The golden Syrian hamster is now considered the gold-standard animal model to study pathogenesis and for antiviral testing, as it is highly susceptible to infection and recapitulates clinical disease symptoms seen in humans (65–67). Similar to SARS-CoV, mice are not naturally susceptible to SARS-CoV-2 infection but can be genetically modified to support viral replication (68, 69). However, some SARS-CoV-2 variants of concern have acquired mutations that enhance binding to mouse angiotensin-converting enzyme 2 (ACE2), rendering wild-type mice partially or fully susceptible to infection (70). Of note, there are significant genetic and phenotypic differences between pre-omicron and omicron SARS-CoV-2 variants, which reflect their evolving virological and clinical characteristics.

For MERS-CoV, continuous circulation of this virus within dromedary camel population likely facilitates zoonotic cross-species transmissions to humans. A comprehensive study reported MERS-CoV seropositivity in dromedary camels in Africa, the Middle East, and Asia (71–74), while horses, sheep, and goats were all seronegative (75). Additionally, the virus can readily replicate in primary camelid airway cultures (76). Although dromedary camels are suspected as the primary zoonotic reservoir for MERS-CoV, several lines of evidence implicate bats as ancestral reservoir hosts. A number of phylogenetically related viral isolates, including BtCoV-HKU4 and BtCoV-HKU5, NeoCoV, and PDF 2180, have been identified in various Vespertilionidae bat species (77). Mice are not naturally susceptible to MERS-CoV infection but can be genetically engineered to support replication (78).

In summary, HCoVs exhibit distinct host ranges: epidemic and pandemic HCoVs are generalists that can infect a variety of different mammals, while endemic seasonal HCoVs are restricted to humans. SARS-CoV and MERS-CoV are not specifically adapted to humans due to recent cross-species transmission events and inefficient human-to-human transmission. In contrast, highly efficient human-to-human transmission allows SARS-CoV-2 to continue to circulate in humans, with a marked reduction in pathogenicity observed as the virus becomes endemic (79). Phylogenetic evidence highlights a likely zoonotic origin for all HCoVs, suggesting potential circulation back and forth between different species, facilitating recombination, adaptation, and potentially driving emergence in novel hosts. Indeed, altered pathogenesis or transmission characteristics could facilitate cross-species transmission events associated with more efficient replication in humans or other mammals. Thus, it is crucial to understand mechanisms both limiting and driving CoV host-switching to prepare for future zoonotic spillover events.

## HOST CELL TROPISM

Successful CoV attachment and subsequent internalization into host cells represent the first stage of the infection process, the first layer of virus-host interactions and a determinant of host, tissue, and cellular tropism. On the virus side, this process is mediated by the envelope-anchored spike glycoproteins. For all CoVs, these glycoproteins are presented as trimeric complexes, which decorate the virion surface and give virions their characteristic “corona” appearance when visualized by electron microscopy (80). These proteins are heavily glycosylated and decorated with N-linked carbohydrate moieties, which are important for entry and immune evasion. CoV spike proteins are type I fusion proteins that form homotrimers (81). Each monomer is typically 1,200–1,400 amino acids in length and consists of three segments—a short intracellular tail, a transmembrane anchor, and a large ectodomain. This ectodomain consists of the receptor-binding S1 subunit and the membrane-fusion-promoting S2 subunit. In addition, the S1 subunit can be further subdivided and contains two independent domains, an N-terminal domain and a C-terminal domain (S1-CTD), both of which can function as receptor-binding domains (RBD) that recognize cell surface molecules (82). Structural studies have revealed that spike proteins undergo extensive conformational changes and structural rearrangements, as well as cleavage, during binding to their cognate receptors. These events precede internalization or virus-host membrane fusion (83–85).

### Attachment factors

Diverse host factors mediate viral attachment to the cell membrane prior to primary receptor engagement. These factors localize the virus near the plasma membrane to facilitate receptor binding and can also induce spike conformational changes. Both HCoV-OC43 and HCoV-HKU1 bind to acidic carbohydrate 9-O-acetylated sialic acid (86, 87). Indeed, sialoglycan binding to the HCoV-HKU1 spike S1 domain triggers conformational changes in spike, which are required for subsequent receptor binding (88, 89). Similarly, MERS-CoV spike binds to sialic acid (90), and HCoV-NL63 spike binds to heparan sulfate proteoglycans (91). In contrast, SARS-CoV-2 attaches to cells via C-type lectin receptors L-SIGN, DC-SIGN, and SIGLEC1 (92). Omicron variants exhibit significantly stronger attachment to host cell membranes, primarily due to increased affinity for the co-receptor heparan sulfate (93). In all cases, attachment factor engagement enhances receptor-mediated entry.

### Primary entry factors

**Fig 2:**
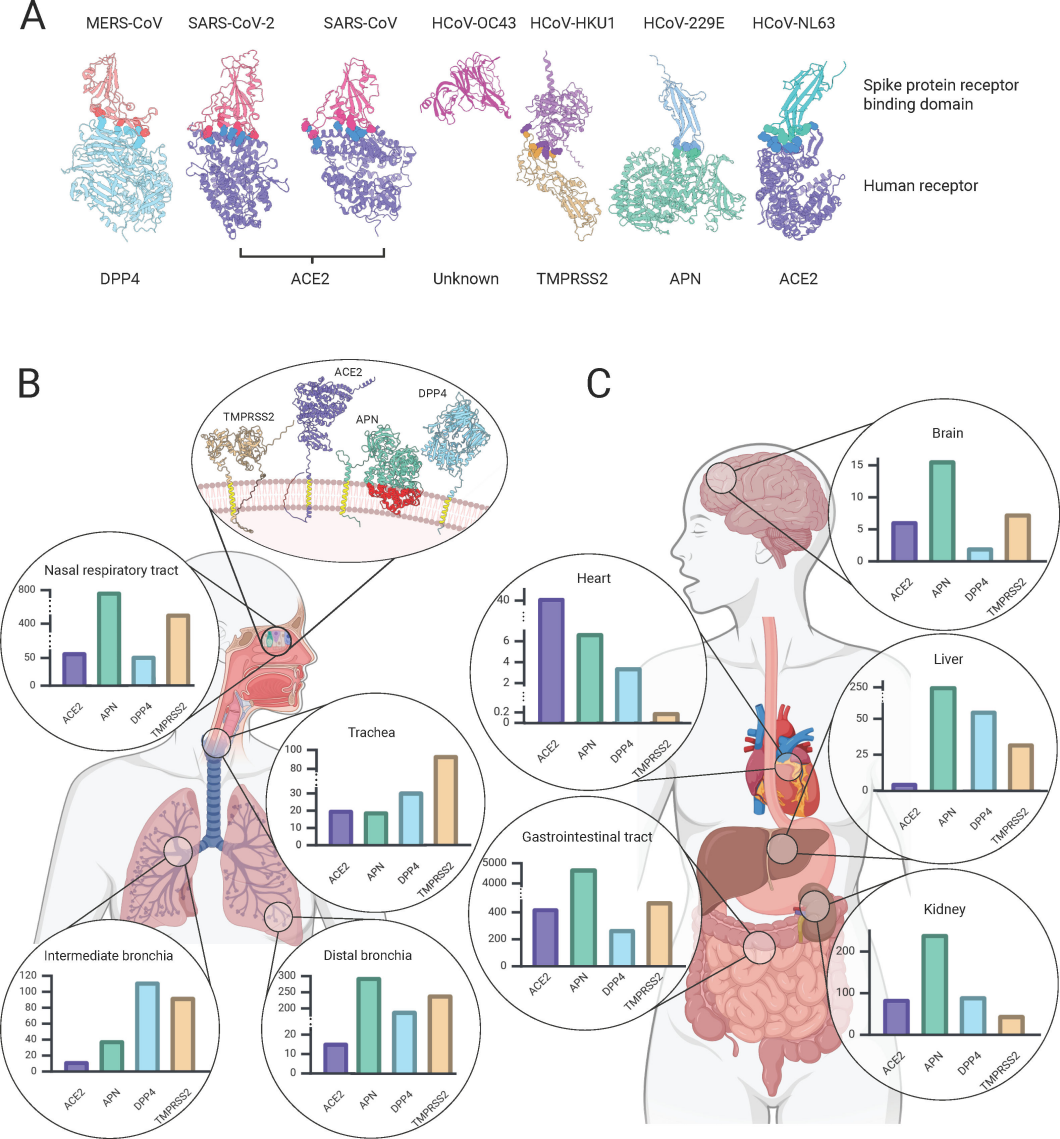
CoV receptor-binding footprints and tissue expression. (**A**) Ribbon diagrams showcase the three-dimensional configurations of the receptor-binding domain of HCoV spike proteins (upper) and depict the interaction with their respective human receptors (lower) (42, 94–98). Structural representations of host receptors were created with AlphaFold and UCSF ChimeraX (99, 100). Spike protein colors match the viruses from Fig. 1A and B, and receptor colors match the following panels. Interacting residues in the binding of spike protein and receptor are depicted in spherical style. (**B**) Illustration of the abundance of receptors for HCoVs within the nasal respiratory tract, trachea, intermediate, and distal bronchia. The anatomical overview highlights primary infection sites, with magnified insets depicting the normalized transcript expression (101, 102). Representations of the human receptors at the cellular level are shown in the top inset and retrieved as described for panel **A** (99, 100). Correctly and incorrectly predicted folding of transmembrane domains are colored yellow and red, respectively. For detailed information on the different receptors and entry factors, as well as a mapping of the individual HCoVs to their respective receptors, see Host Cell Tropism. (**C**) Normalized transcript expression for secondary infection sites is presented as described in panel **B** (103). Brain data are comprised from hippocampal formation, amygdala, basal ganglia, midbrain, spinal cord, cerebral cortex, cerebellum, hypothalamus, and choroid plexus, and gastrointestinal data from stomach, small intestine, colon, rectum, and duodenum. The figure was created with BioRender.com.

To date, four distinct plasma membrane-anchored enzymes have been identified to act as primary receptors for human endemic, epidemic, and pandemic CoVs (Fig. 2A). Of note, all four described CoV receptors are proteolytically active. However, this protease activity is not required for successful receptor engagement. Indeed, CoV spike proteins bind to proximal regions of these proteases with minimal disruption of enzymatic activity.

#### Angiotensin-converting enzyme 2

HCoVs -NL63, SARS-CoV, and SARS-CoV-2 all engage ACE2 as their primary receptor to facilitate cell entry (60, 104–106). ACE2 is a heavily glycosylated type I transmembrane protein and is part of the renin-angiotensin system, regulating blood pressure and electrolyte balance through coordinated effects on the heart, blood vessels, and kidneys (107). The ACE2 ectodomain has a zinc-dependent carboxypeptidase, which hydrolyzes angiotensin II to form Ang-(1–7), promoting vasodilation and inhibiting fibrosis and inflammation (108).

Cryo-EM studies of authentic SARS-CoV-2 virions indicate that spike proteins form trimers with two flexible hinges in the stalk domain, enabling cell surface scanning to locate receptor complexes and initiate cell entry (80). A cryo-EM structure for the SARS-CoV-2 spike trimer in a prefusion conformation confirms the three RBDs are located at the head of the globular trimer and show a single RBD in an “up” conformation, ready for receptor engagement (109). The crystal structure of the SARS-CoV RBD bound to the peptidase domain of human ACE2 highlights binding to the N-terminal lobe of the peptidase (94). Interestingly, trimer structures and RBD engagement of ACE2 for SARS-CoV and SARS-CoV-2 are similar, although SARS-CoV-2’s binding affinity for ACE2 is reportedly 10–20-fold higher than for SARS-CoV (109). A distinguishing feature of the omicron variant is its increased binding affinity to the human ACE2 receptor compared to the wild-type virus, which is attributed to multiple mutations in the RBD of the spike protein (110). The SARS-CoV-2 RBD, located in the S1 domain of spike, is composed of five stranded antiparallel betasheets, stabilized by four pairs of disulfide bonds. Crystallographic comparison of SARS-CoV and SARS-CoV-2 RBDs: ACE2-binding interfaces revealed nonidentical but overlapping binding profiles. Of the 20 ACE2 residues that interact with either the SARS-CoV or SARS-CoV-2 RBDs, 17 residues are shared between both interactions (94). These similar binding profiles likely reflect shared inheritance from a common ancestor rather than convergent evolution, as both viruses are genetically divergent, but likely ACE2 receptor usage is a conserved property among their distinct progenitor lineages (94). HCoV-NL63 similarly utilizes ACE2 as its cellular receptor. While the S1 domains of HCoV-NL63 and SARS-CoV are quite different, they both associate with a region of human ACE2 that includes a key loop formed by beta strands 4 and 5. However, the S protein interaction of HCoV-NL63 with ACE2 is uniquely sensitive to residue 354, unlike the S protein of SARS-CoV, which is only modestly affected. This suggests that while both viruses bind overlapping regions of ACE2, HCoV-NL63 relies more heavily on the specific conformation or interactions for efficient receptor binding (111).

High sequence homology of SARS-CoV and SARS-CoV-2 has been described for several bat isolates. SARS-CoV-like viruses, RsSHC014 and Rs3367, were recovered from Chinese horseshoe bats, and bat virus SL-CoV-WIV1, isolated from bat fecal samples and propagated on VeroE6 cells, was able to utilize bat, civet, and human ACE2 for cell entry (54) (please refer to Species Tropism). ACE2 orthologs from 46 bat species exhibit different levels of interaction with the spike proteins of SARS-CoV and SARS-CoV-2. ACE2 orthologs from 24, 21, and 16 bat species were resistant to infection with either SARS-CoV, SARS-CoV-2, or both viruses, respectively, indicating not all bat species can act as hosts for these viruses (112). ACE2 orthologs from a further 48 mammalian species, including domestic animals, pets, and zoo animals, were also investigated for their ability to bind SARS-CoV-2 spike (beta variant [B.1.351]), with 44 species supporting viral entry (113). Of note, mutations at residues H41 and E42 in ACE2 orthologs from New World monkeys disrupt their ability to bind the viral spike protein and explain their resistance to SARS-CoV-2. Viruses closely related to SARS-CoV-2 are also reported to circulate in wild pangolins, which are predicted to bind to human and pangolin ACE2 (114). These animals are heavily trafficked and therefore represent a risk for zoonotic spillover events. Indeed, subsequent studies have confirmed that pangolin CoVs can utilize human ACE2 as a receptor and can cause severe disease in K18-hACE2 transgenic mice (115, 116). A novel mink respiratory CoV also utilizes ACE2 as a receptor and can additionally utilize bat, monkey, and human orthologs to enter cells, binding to the same interface as SARS-CoV-2 (117).

Initially, merbecoviruses were considered to exclusively utilize dipeptidyl-peptidase 4 (DPP4) to initiate cell infection (see below). However, multiple independent studies have identified a range of merbecoviruses that can use ACE2 for cell uptake. Bat CoVs PDF-2180 and NeoCoV, close relatives of MERS-CoV, reportedly bind efficiently to bat ACE2 (and less efficiently, human ACE2), and cryo-EM identified distinct noncanonical ACE2:RBD binding interfaces which involve protein-glycan interactions (118). Two European bat CoVs related to MERS-CoV, MOW15-22 and PnNL2018bb, engage ACE2 through distinct surface-binding regions but cannot bind human ACE2 and exhibit a narrow species range (119). The related merbecovirus HKU5 also enters cells of *Pipistrellus* bats and other mammals via ACE2 engagement (119), and a novel lineage 2 isolate, HKU5-CoV-2, was also able to infect human-ACE2 expressing cells (120). These studies highlight ACE2 utilization evolved independently on multiple occasions among both sarbecoviruses and merbecoviruses.

Together, these investigations highlight high promiscuity and broad ACE2 receptor usage for divergent endemic, epidemic, pandemic, and nonhuman CoVs. These data also underscore the requirement for continued virological surveillance—including systematic sampling, sequencing, and characterization—of ACE2 utilizing CoVs in wild bats, farmed mink, and heavily trafficked species such as pangolins. While such efforts may not directly lead to vaccines being stockpiled in advance, they enable early risk assessment, guide targeted virological and ecological studies, inform policies on wildlife trade and farming practices, and allow for the rapid development of diagnostics, antivirals, and vaccines based on pre-existing knowledge.

#### Aminopeptidase N

Aminopeptidase N (APN; a.k.a. CD13) is the primary receptor used by HCoV-229E to enter cells (121). APN represents an ~150 kDa type II transmembrane protein, which exhibits extensive N-linked glycosylation and contains a zinc-containing aminopeptidase ectodomain. APN plays a role in a range of physiological processes, including pain sensation, blood pressure regulation, tumor angiogenesis and metastasis, immune cell chemotaxis, sperm motility, and cell adhesion (122).

X-ray crystallographic and cryo-EM studies indicate that HCoV-229E spike RBD:APN receptor binding is mediated by the interaction of three RBD loops that are located in the S1-CTD (95, 123, 124). The RBD must flip to an “up” position to engage APN (95). Phylogenetic analysis of RBD loops from deposited HCoV-229E database sequences identified six RBD classes, with RBD sequences representing the most variable regions of the viral genome. Indeed, conformational plasticity in these regions drives differences in APN-binding affinity and antibody recognition observed between circulating strains (123).

APN orthologs from multiple species also serve as receptors for pathogenic CoVs infecting nonhuman species, including transmissible gastroenteritis virus (TGEV—infecting pigs) (125), feline infectious peritonitis virus, and feline enteric CoV (126). Studies of chimeric human, porcine, and feline APN glycoproteins reveal APN receptor and species specificity is determined by two crucial regions (127–129), with differences in N-linked glycosylation of APN also representing important determinants of species range (123, 127). These studies confirm broad APN receptor usage by distinct alpha-CoVs with different routes of transmission and associated pathology—HCoV-229E has relatively mild respiratory symptoms in humans, while TGEV infects the gastrointestinal tract and causes fatal diarrhea in piglets. Combined, these studies highlight the potential for cross-species transmission of highly pathogenic animal CoVs to humans based on shared APN receptor usage, although barriers and incompatibilities exist.

#### Dipeptidyl-peptidase 4

DPP4 (a.k.a. CD26) is the primary receptor used by MERS-CoV for cell entry (130). DPP4 is a type II transmembrane glycoprotein with a short cytoplasmic tail and a large extracellular region composed of a short flexible segment, separate glycan-rich and cysteine-rich domains, and a C-terminal serine-protease domain with catalytic activity that mediates cleavage and inactivation of a range of circulating regulatory peptides (131).

Crystallographic and cryo-EM studies have provided structural insights into MERS-CoV spike RBD:DPP4 binding, identifying domains, interfaces, and critical residues involved in this interaction (94, 132). A 3.0 Å-resolution crystal structure of the MERS-CoV RBD in complex with soluble DPP4 reveals that the RBD directly interacts with the beta-propeller domain of DPP4 through two major patches, which do not overlap with the C-terminal protease domain (96). Cryo-EM structures of prefusion trimeric MERS-CoV spike in complex with DPP4 reveal the RBD can occur in two states: “standing” or “lying.” This dynamic and flexible RDB contributes to efficient DPP4 recognition. Only “standing” RDBs on monomeric spike trimer subunits can bind to DPP4 (132).

MERS-CoV likely originates from bats, with DPP4 orthologs from 16 bat species supporting MERS-CoV infection to varying degrees (133). Serial passage of MERS-CoV on cells expressing a suboptimal bat DPP4 variant resulted in the accumulation of spike mutations that boost entry, highlighting the rapid adaptability of MERS-CoV to improve virus-host receptor interactions which can occur by multiple mutational pathways (133). MERS-CoV cannot utilize murine, hamster, or ferret DPP4 for cell entry (134, 135). Despite its zoonotic origin, MERS-CoV spike preferentially binds to human DPP4 over its bat ortholog to enter cells, while bat CoV HKU4-CoV can only efficiently utilize bat DPP4 (136). A bat MERS-like CoV similar to HKU4-CoV circulates in Malayan pangolins and binds to human, bat, and pangolin DPP4. This MjHKU4r-CoV-1 virus is infectious in human airway and intestinal organoids, and pathogenic in hDPP4 transgenic mouse lungs (137). Furthermore, Ty-BatCoV HKU4 was isolated from lesser bamboo bats and was shown to utilize DPP4, with replication and cytopathology reported in human cells and hDPP4 transgenic mice (138). Together, these studies demonstrate the rapid adaptability of MERS-CoV to novel host DPP4 utilization and the circulation of multiple DPP4-utilizing viruses in both bats and rodents, highlighting the potential for future spillover events to humans and the requirement for continued surveillance.

#### Transmembrane protease serine 2

TMPRSS2 serves as a functional receptor for HCoV-HKU1, required for both viral entry and spike-mediated cell-cell fusion (41). TMPRSS2 is a type II transmembrane protein with a cytoplasmic tail domain. The large extracellular portion contains low-density lipoprotein receptor class A domain, a scavenger receptor cysteine-rich domain, and a C-terminally encoded serine protease domain (139). The serine protease activity is not required for HCoV-HKU1 entry, and catalytically inactive TMPRSS2 can still facilitate HCoV-HKU1 entry via an endosomal route (41). In addition to its role as an entry receptor for HCoV-HKU1, TMPRSS2 is also involved in proteolytic priming of diverse CoV spike glycoproteins to enhance viral uptake.

Crystallographic and cryo-EM studies have determined the virus:host molecular interaction interface at high resolution (42, 89, 140, 141). Zymogenic TMPRSS2 undergoes autocleavage to become fully active, inducing conformational changes in three activation loops that increase HCoV-HKU1 RBD-binding affinity (141). The crystal structure of the HCoV-HKU1 RBD-TMPRSS2 complex highlights that in trimeric spike, at least one RBD must be in a “up” confirmation to allow binding to the periphery of the catalytic groove of the TMPRSS2 serine protease domain (141). Cryo-EM studies further confirm sialoglycan binding induces conformational changes that promote RBD opening, enabling spike binding to TMPRSS2 via multiple key residues and interfaces (42, 89). HCoV-HKU1 utilizes glycan shielding and conformational masking to evade host humoral responses while maintaining TMPRSS2 engagement, illustrating general immune evasion strategies that can complicate vaccine design for HCoVs (42).

HCoV-HKU1 genotypes A and C share a highly conserved mechanism for sequential binding of sialoglycans and TMPRSS2, suggesting a universal mode of receptor recognition across different circulating strains (89). TMPRSS2 residues D417 and Y469 are reported as critical for human specificity and determinants of the narrow host range of HCoV-HKU1 (141, 142). Despite this restricted tropism, the HCoV-HKU1 RBD footprint of TMPRSS2 contact residues is largely conserved among diverse mammals. Indeed, HCoV-HKU1 spike-mediated cell entry is facilitated by TMPRSS2 orthologs from distinct mammalian orders, including primates, rodents, artiodactyls, lagomorphs, and bats (42) (please refer to Species Tropism). This RBD footprint is only partially conserved in both reptiles and birds and minimally conserved in amphibians and other vertebrates. These observations support recent findings indicating cell entry is not the major barrier which limits viral cross-species transmissions, with downstream incompatibilities in novel host cells representing a major obstacle (143, 144).

### Accessory proteases

#### Furin

Furin is a ubiquitously expressed calcium-dependent serine protease, which localizes in the Golgi apparatus where it cleaves precursor proteins at their basic amino acid processing site into mature or active forms. A polybasic (PRARR) insertion in the SARS-CoV-2 S1/S2 spike junction allows proteolytic processing by furin during virion egress from infected cells (105). Furin cleavage at S1/S2 exposes the C-terminus of S1 and facilitates subsequent spike binding to neurophilin-1, which ultimately primes the spike protein for enhanced host cell infection. This is specific for SARS-CoV-2 (145, 146). Indeed, the presence of the furin cleavage site in SARS-CoV-2 confers a selective advantage in lung cells, human airway epithelial cultures, and is required for efficient ferret-to-ferret transmission (84), while furin inhibition has been shown to suppress spike-mediated syncytia formation in SARS-CoV-2-infected cells (147). Still, evolutionary analysis of diverse CoV spike proteins suggested that furin cleavage motifs have arisen independently on multiple occasions in the family Coronaviridae (148). Omicron carries three mutations close to the furin cleavage site (P681H, H655Y, and N679K), which reduce the efficiency of spike protein cleavage at the S1/S2 junction (149). Furin is also co-opted by unrelated viruses, including mosquito-transmitted orthoflaviviruses, where the chaperone protein prM is cleaved by furin at a conserved polybasic motif to facilitate envelope dimerization and virion maturation during particle morphogenesis (150).

#### TMPRSS2 and cathepsin L

After receptor engagement, which induces conformational changes in the spike protein, there are two distinct cellular locations at which CoV-host membrane fusion can potentially occur: at the cell surface or in endosomal compartments. TMPRSS2 is localized to the plasma membrane, and co-opting of TMPRSS2 to enhance virus infection was initially described for influenza, where hemagglutinin cleavage for proteolytic activation was demonstrated (151). Subsequently, TMPRSS2 was shown to be a broad enhancer of CoV infection, augmenting uptake into permissive cells of SARS-CoV (152), HCoV-229E (153), MERS-CoV, and SARS-CoV-2 (154–157). The abundant expression of TMPRSS2 in small intestinal enterocytes and hepatocytes contributes to SARS-CoV-2 tropism for intestinal cells and the liver, while the ability to enhance SARS-CoV-2 entry is conserved among TMPRSS2 orthologs from diverse mammalian orders (154, 156, 157).

TMPRSS2’s enzymatic activity cleaves spike at S2′ to expose the fusion peptide and facilitate membrane fusion. TMPRSS2 infection enhancement is dependent on its serine protease activity, which can be blocked pharmacologically or by mutational deletion of the HDS catalytic triad (157, 158). In the presence of TMPRSS2, the SARS-CoV-2 spike protein is proteolytically cleaved at S2′ at the cellular surface. Under these conditions, the virus enters the cell within 10 minutes in a pH-independent manner, bypassing the endosomal route (159). If the cell expresses insufficient amounts of TMPRSS2, the entire virus-receptor complex is internalized via clathrin-mediated endocytosis (160) into endolysosomes, where spike cleavage is mediated by CTSL, a cysteine protease that functions in protein catabolism and requires an acidic environment for proteolytic activity (159, 161). This step takes up to 60 minutes post infection. Therefore, TMPRSS2 expression levels are proposed to determine which route the virus utilizes to enter ACE2-expressing cells, with TMPRSS2-primed entry more efficient than endosomal CTSL priming. More recently, EM visualization and quantification of early SARS-CoV-2 entry steps showed TMPRSS2-mediated enrichment of internalized virions into endosomal compartments, which was unexpected and requires further investigation (157). Together, the dual functions of TMPRSS2, serving as both a primary receptor and entry enhancing co-factor, highlight its importance for CoV entry, in general, and its potential as a therapeutic target.

While numerous post-entry factors can influence the efficiency of CoV replication, these alone are generally insufficient to determine cellular susceptibility. For many viruses, cellular entry is not the primary barrier to productive infection. However, this does not appear to apply to CoVs, for which the presence and availability of the appropriate entry receptor remain the dominant determinants of host cell permissiveness (124). This underscores the unique reliance of CoVs on receptor-mediated entry as a critical gatekeeper of infection. Follow-up studies will likely reinforce these findings, providing further insights into the essential role of entry receptors in CoV infectivity.

### Suspected additional receptors

While bona fide receptors for human CoVs are well characterized and undisputed, multiple additional proteins have been identified as potential receptors or entry co-factors, mostly for SARS-CoV-2. Examples include glucose-regulated protein 78, a molecular chaperone, which was proposed as an auxiliary factor that facilitates SARS-CoV-2 entry by forming a complex with the spike protein and ACE2 (162, 163). Additionally, the receptor tyrosine kinase AXL was identified as a candidate receptor that promotes infection of pulmonary and bronchial epithelial cells, while the glutamyl-aminopeptidase was proposed as a co-receptor due to its co-expression with ACE2 in various tissues (164, 165). Angiotensin-II receptor type 2 (AGTR2, a G protein-coupled receptor) and basigin/CD147 were initially described but are now considered unlikely candidates for SARS-CoV-2 receptors. Confirmation of AGTR2 as an entry factor was not supported by additional studies (166), and the role of CD147 remains unclear, with conflicting data on its ability to mediate viral entry (167–169). Similarly, TMEM106B was initially described as a proviral host factor for SARS-CoV-2 (170) and subsequently shown to bind directly to the spike RBD and mediate ACE2-independent entry into cells (171). However, more recently, while TMEM106B-mediated infection was confirmed to mediate ACE2-independent entry *in vitro*, this phenotype could not be recapitulated *in vivo* (172). In summary, these findings highlight the potential complexity of the CoV-host interactome during the entry process but also underscore the need for further basic research to validate these proteins in the CoV infection process before definitive roles can be assigned. In addition, CoV utilization of nonhuman orthologs in susceptible reservoir species should also be confirmed.

## TISSUE TROPISM

HCoVs are primarily recognized as respiratory pathogens and mainly transmitted via respiratory droplets and aerosols, making the respiratory tract the first and most common site of viral entry (173). Virions are inhaled into the upper and lower respiratory tracts, encountering epithelial cells lining the nasal passages, throat, and lungs. Consequently, the respiratory system serves as the primary gateway for HCoV infection, facilitating viral replication and the subsequent spread to other tissues and organs (174). Of note, pathogenic SARS-CoV, MERS-CoV, and SARS-CoV-2 can be disseminated systemically, affecting multiple organs beyond the lungs (Fig. 2B and C). The predominance of apical-only release for SARS-CoV and SARS-CoV-2 suggests these viruses are unlikely to breach the epithelial barrier and enter circulation. In contrast, basolateral release, as seen with MERS-CoV (and certain animal CoVs like mouse hepatitis virus, canine CoV, and feline CoV), is associated with systemic spread. Incorporating these distinctions can clarify why some CoVs remain localized while others may disseminate (175). It is important to note that the evidence of coronavirus presence in extrapulmonary tissues primarily comes from *in vitro* studies or post-mortem analyses of patients with severe disease, often complicated by underlying conditions. Thus, such findings may be artificial or reflect late-stage dissemination facilitated by tissue damage and may not represent typical viral tropism during average infections, underscoring the need for cautious interpretation.

### Respiratory tract

Most HCoVs begin their infection in the nasal epithelium, where the virus first establishes itself. Figure 2 illustrates the expression profiles of host receptors that mediate binding of the seven HCoVs within tissues of the nasal, pharyngeal, and distal respiratory tract. Endemic HCoV-229E primarily infects non-ciliated cells, whereas HCoV-OC43, -NL63, and -HKU1 preferentially infect ciliated cells in the respiratory tract (176–178). HCoV-HKU1 has been additionally shown to infect primary human alveolar type II cells (13). HKU1 recognizes TMPRSS2, facilitating viral entry, which is highly expressed in small airway epithelium and nasal epithelium in contrast to a lower expression in masticatory mucosa (179), as illustrated in Fig. 2B, potentially contributing toward a preferential cellular susceptibility within the nasal mucosa. Interestingly, HCoV-NL63 utilizes ACE2 for cell entry, like the more pathogenic SARS-CoV and SARS-CoV-2. Thus, it shares some of the cellular tropism; however, HCoV-NL63 usually induces only mild or moderate respiratory disease. Clinical data indicate that immune control could partly determine pathogenicity, as infants and immunocompromised individuals experience more severe disease with HCoV-NL63 (37, 177, 180). Additionally, differences in ACE2 interactions could contribute to differential pathogenicity (106). ACE2 expression is higher in the nasal epithelium, gradually decreasing toward the lung, influencing tropism (181, 182) (Fig. 2B). Infection with SARS-CoV and SARS-CoV-2 can cause significant damage to the lung epithelium. This, combined with a heightened inflammatory response, often leads to alveolar injury and the development of acute respiratory distress syndrome, which can progress to respiratory failure (183, 184). Within the respiratory system, SARS-CoV and SARS-CoV-2 have been shown to infect ciliated epithelial cells, goblet cells, and alveolar type II cells (113, 185, 186). MERS-CoV similarly primarily infects tissues of the respiratory tract. The virus enters human cells via interaction with the DPP4 receptor, which is widely expressed on the surfaces of respiratory epithelial cells, particularly in the lungs (187), as illustrated in Fig. 2B. On a cellular level within the respiratory tract, specifically goblet cells have been shown to support viral replication (188). The clinical spectrum can range from asymptomatic or mild respiratory disease to severe pneumonia including ARDS but also multiorgan failure (189, 190).

### Central nervous system

All seven human-infecting CoVs have been associated with central nervous system (CNS) dysfunctions (191). Reports of encephalitis associated with seasonal hCoVs are rare and largely anecdotal and are often limited to immunocompromised or pediatric patients. For example, HCoV-229E has been described to have the capacity to infect neuroblastoma, neuroglioma, and oligodendrocytic cells, which is likely related to the high expression of its receptor aminopeptidase N in the brain (192) (Fig. 2C). HCoV-OC43 is a neurotropic virus that can invade the central nervous system and primarily infects neurons, as has been demonstrated in both human and mouse models. Infection of neurons can lead to neurodegenerative effects that contribute to cell stress and apoptosis. This neuroinvasive ability has been associated with neurological complications, such as encephalitis and neurodegenerative diseases (34, 35). HCoV-NL63 also appears to be neurotropic in very rare cases. The virus is able to infect mononuclear circular cells, and few independent cases of encephalopathy are reported (193, 194). ACE2 is expressed by neuronal and glial cells in the brainstem. Expression has also been detected in the amygdala and cerebral cortex, with the highest levels observed in the pons and medulla oblongata (195, 196). The more pathogenic hCoVs have been more clearly associated with neurological complications; however, even for these viruses, encephalitis remains a rare complication. For SARS-CoV-2, neurological manifestations were reported, including encephalopathy and anosmia (loss of smell), suggesting that SARS-CoV-2 can affect the CNS (197, 198). Compared to SARS-CoV, SARS-CoV-2 is associated with a broader spectrum and greater frequency of neurological symptoms. This may be due to its higher affinity for ACE2, as described in Host Cell Tropism,’ and the additional use of co-receptors such as neuropilin-1, which is highly expressed in nervous tissue (199). SARS-CoV-2 also has a greater neuroinvasive potential, possibly entering the CNS via the olfactory nerve, hematogenous spread, or infected immune cells (200, 201). In addition, it triggers greater systemic inflammation and endothelial dysfunction, contributing to cerebrovascular complications (202). High levels of DPP4 protein have been observed in the fetal and perinatal human brain, particularly within neuroblasts, neurons, brain capillaries, the ependymal lining, and the choroid plexus. In adulthood, however, DPP4 mRNA expression in the brain is markedly lower, especially when compared to organs such as the placenta, kidneys, lungs, and liver (191). Neurological involvement in MERS-CoV infections is rare but has been reported in a small number of cases, with symptoms ranging from confusion to encephalitis. However, the precise mechanism underlying these pathways remains incompletely understood.

### Liver, kidney, and gastrointestinal tract

HCoV-229E replicates in Huh7 cells, an immortalized human liver cell line commonly used as the standard for infection experiments with this virus (203). *In vivo*, however, there is no evidence of liver tropism. The virus is also associated with gastrointestinal symptoms, including abdominal pain, diarrhea, and vomiting. These symptoms are thought to be due to the ability of the virus to infect next to respiratory epithelial cells and also intestinal epithelial cells. It should be mentioned that the presence of viral RNA in stool could reflect passive transit rather than local replication, unless supported by additional evidence such as detection of viral proteins, infectious virus, or histopathological findings from gastrointestinal tissue. Virus particles resembling HCoV-OC43 have been detected in stool samples from infected patients, suggesting active replication in the intestinal mucosa (204). HCoV-HKU1 has also been detected in stool samples, and the virus is associated with symptoms of the gastrointestinal tract (205). In cell culture, HCoV-HKU1 is very different from the other HCoVs. It has not yet been possible to grow the virus in immortalized cell lines; only cultivation in primary human respiratory epithelial cell cultures has been successful (206). For HCoV-NL63, viral RNA has also been detected in a few stool samples from children with acute gastroenteritis (204, 207). In the laboratory, LLCMK2 cells, an immortalized cell line derived from epithelial cells of primate kidney, are used to propagate this virus (208). A recent study demonstrated differential susceptibility and replication kinetics for HCoV-229E, -NL63, and -OC43 in various cell lines, with HCoV-OC43 alone being able to replicate in extra-pulmonary tissues, including human colon cancer cells and African green monkey kidney cells (209). It has been shown that SARS-CoV RNA is not only found in the lungs, bronchi, and trachea but also in the stomach, small intestine, sweat glands, pancreas, liver, and adrenal glands (210, 211). Indeed, a high density of ACE2 receptors has been described within the kidneys and gastrointestinal tract (212) (Fig. 2C). In addition, *in situ* hybridization implicated the presence of the virus in various tissues, specific in the epithelial cells of mucosa of the small and large intestine, in the stomach and in the esophagus of the digestive tract, in the distal tubular epithelium within the kidney, and hepatocytes in the liver (185). SARS-CoV-2 has been shown to affect the kidneys, which can lead to an acute kidney injury. ACE2 is expressed on proximal tubules, parietal epithelial cells, mesangial cells, and podocytes (213). Also, gastrointestinal involvement is a recognized feature of SARS-CoV-2 infection. The virus can infect enterocytes via ACE2, which is highly expressed in the small intestine. Possible symptoms include diarrhea, nausea, vomiting, and abdominal pain, which may occur even in the absence of respiratory manifestations. Viral RNA has been detected in fecal samples, suggesting active replication in the gastrointestinal tract and possible fecal-oral transmission, although the latter remains under investigation (214, 215). Liver dysfunction, including elevated liver enzymes, has been observed specifically after SARS-CoV-2 infection, especially those with severe COVID-19 disease. Although ACE2 is modestly expressed in this tissue, it is sufficient to enable productive replication (154, 216, 217) (Fig. 2C). MERS-CoV has also been found to affect other organs, although less frequently reported compared to SARS-CoV-2. The presence of DPP4 receptors in tissues, such as the kidneys, gastrointestinal tract, and liver, allows the virus to spread beyond the lungs (218, 219). Renal failure has been observed in some severe cases, particularly in patients with pre-existing conditions (220). MERS-CoV has also been associated with mild gastrointestinal symptoms, including diarrhea, although this is less common (221), and human intestinal cells have been experimentally shown to be susceptible to infection, prompting speculation about the human intestinal tract as an alternative infection route (222).

### Circulatory system and heart

The human heart is characterized by particularly high ACE2 expression, especially in cardiomyocytes, endothelial cells, and pericytes, highlighting its potential susceptibility. In patients with or without a history of cardiovascular disease, cardiac function may be impaired in the context of SARS-CoV-2 infection (223, 224) (Fig. 2C). SARS-CoV-2 has been found in myocardial tissue, which can possibly lead to cardiovascular manifestations, such as myocarditis, pericarditis, acute coronary syndrome, thromboembolic events, and heart failure (223, 225). Cardiovascular complications were occasionally reported in SARS-CoV infection, but data remain limited and largely anecdotal due to the lack of systematic studies. Case reports and small cohorts described events such as acute myocardial infarction, transient diastolic dysfunction, tachycardia, hypotension, and rare arrhythmias, most of which were self-limiting and occurred in otherwise asymptomatic patients (226). Autopsy findings from a small study revealed thromboembolic events and myocardial infarction, though the relevance of these findings remains unclear due to small sample sizes and lack of confirmatory studies (227). Although DPP4 is widely expressed in the vascular system, the available data on cardiac tropism of MERS-CoV are very limited (228). To date, there is little direct evidence supporting significant involvement of the heart in MERS-CoV infection, and dedicated studies addressing this aspect are lacking (229). For endemic HCoVs, cardiovascular effects are rare and typically limited to exacerbation of pre-existing heart conditions in vulnerable individuals (227).

The exact mechanisms by which SARS-CoV-2 and other HCoVs spread to different organs are still unclear. It is suspected that the virus may reach distant tissues through the bloodstream or could be transported on immune cells. Another theory suggests that nerve pathways could facilitate viral spread (200, 230–232).

In conclusion, experimental and clinical evidence suggests a broad tissue and cell tropism associated with both epidemic and pandemic HCoVs, affecting the hematological, cardiovascular, renal, gastrointestinal and hepatobiliary, endocrinological, neurological, ophthalmological, and dermatological systems (233). In addition to receptor availability, additional factors, including co-opted host factors, immune modulation, and the role of specific cellular signaling pathways, may contribute to HCoV spread and tissue tropism, highlighting the need for further research to fully determine the profiles of permissive and susceptible cells (234, 235).

## CONCLUSIONS

It is estimated that more than 60% of emerging viruses causing disease in humans originate from zoonotic transmission (236). CoVs represent a large family of viruses associated with a wide range of diseases, including respiratory, enteric, hepatic, and neurological manifestations and have been detected in a wide range of vertebrate species (4). It is important to note that the interpretation of CoV tropism is highly dependent on the experimental model used, with each system (ranging from immortalized cell lines to organoids and animal models) offering distinct advantages and limitations. Careful selection and contextual understanding of these models are essential to accurately assess viral behavior and improve the translation of findings to human physiology and disease.

Despite significant advances in our understanding of HCoVs, critical gaps in knowledge remain. The precise mechanisms underlying viral persistence, particularly in asymptomatic or mildly symptomatic individuals, are not fully elucidated. Similarly, host restriction and dependency factors that govern species specificity and transmission dynamics across seasonal HCoVs are understudied and still poorly defined. Moreover, a deeper understanding of cross-reactive immunity—especially how prior exposure to seasonal HCoVs may influence responses to emerging pathogens like SARS-CoV-2—is urgently needed.

Progress in these areas is hampered by several technical limitations. The lack of robust *in vitro* models for certain HCoVs continues to constrain experimental validation of host-virus interactions. Moreover, primary human airway cultures and organoids, which are essential for physiologically relevant studies, remain limited in availability and standardization and lack important immune factors. Research tools and genomic databases also exhibit a notable underrepresentation of non-SARS HCoVs, limiting cross-comparative analyses and mechanistic insight. Overcoming these barriers will require interdisciplinary collaborations and coordinated infrastructure to support both basic and translational research. Together, these efforts will be crucial for anticipating and mitigating future coronavirus emergence events.

Many factors are predicted to increase zoonotic spillovers from wildlife to humans in the future, driven by increasing human encroachment into wildlife habitats and accelerating climate change (237–239). The likely zoonotic origins of HCoVs underscore that cross-species spillover events have been central to the emergence of human pathogens. HCoVs typically cause respiratory infections ranging from mild common cold symptoms (as seen with seasonal HCoVs) to severe disease, such as pneumonia and acute respiratory distress syndrome, exemplified by SARS-CoV, MERS-CoV, and SARS-CoV-2. The rapid adaptation of SARS-CoV-2 to humans, including its efficient utilization of the ACE2 receptor and TMPRSS2 protease for entry, highlights how quickly CoVs can evolve to establish sustained human-to-human transmission. Given these dynamics, monitoring zoonotic reservoirs remains critical—not only to detect viruses already using key human receptors such as ACE2, APN, DPP4, or TMPRSS2, but also to identify emerging variants with pandemic potential before widespread human infection occurs. For example, CoVs closely related to SARS-CoV and MERS-CoV that utilize ACE2 or DPP4 receptors pose the highest risk of spillover and adaptation. Targeted surveillance in animal hosts, combined with functional studies of viral tropism and receptor usage, can inform risk assessment and guide early intervention strategies. By deepening our understanding of CoV host adaptation, tropism, and receptor interactions, we can improve pandemic preparedness and response efforts. This proactive approach will help mitigate the global impact of emerging CoVs and reduce the likelihood of future pandemics by enabling timely identification and containment of novel threats. Importantly, addressing these gaps will require sustained efforts in field surveillance, viral genome cataloging, and functional characterization of novel coronaviruses in both known reservoirs (e.g., bats) and high-risk interfaces such as wildlife trade and wet markets. Bats, for example, may serve as key reservoirs for coronavirus evolution, similar to the role of birds and pigs in influenza (240). Evidence of spike gene recombination among bat coronaviruses supports their potential to generate viruses with altered host tropism (241).

A particular focus on receptor-dependent and -independent mechanisms of viral tropism, combined with intensive wildlife surveillance, is required to identify potential host shifts to humans before they occur. Understanding these molecular interactions at the organismal level will help predict which CoVs are most likely to cross species barriers and establish sustained human-to-human transmission.

Finally, improved understanding of CoV tropism has significant implications for the development of vaccines and therapeutics. By elucidating the mechanisms governing tissue and species specificity, such insights can inform the design of broadly protective or universal interventions that target conserved viral entry pathways. This knowledge could ultimately enhance preparedness against both current and emerging CoV threats by enabling more effective cross-reactive immune responses.
